# Mother and Offspring

**Published:** 1890-11

**Authors:** M. F. Cusack


### MOTHER AND OFFSPRING.

BY MISS M. F. CUSACK, IN "GOOD HEALTH."

Everywhere the same old story : poor little mortals, playing and laughing—and, it must be admitted, crying, also, once in awhile. They think not at all of the future, when there may be so many tears in store for them. And the mothers ? Well, it seems to me, sometimes, the mothers think as little about the future of their children as the children do. But the babies have no knowledge of their future life, and the mothers have. Will there not be a good deal of responsibility somewhere ? Will not God say one day to the mothers, "I gave you these little ones to care for ; what did you do to prepare them for their future in time or in eternity ?" It is not that these mothers do not care for their children—on the contrary, they love them dearly ; but they do not always give them wise care. We have had mothers'

meetings and talks to mothers, and much has been said that would have done these young mothers so much good if they had only heard it. But I noticed with pain that at all of these meetings the young mothers who should have been there were absent.

The young mothers, I fear, think they know every thing. The old mothers are just beginning to find out how little they know. But these old mothers—grandmothers, many of them—had evidently begun at the beginning with their own children, and were reaping now the harvest ripe from early sowing—happy mothers and happy children.

If mothers would only think seriously of their responsibility to their children while they are young, they would not so often have to mourn over their short-comings or their sins in later life. I think one cause of the mother's neglect of the mental and spiritual education of children, is that few mothers remember at what a very early age a child "takes notice." The very best of mothers are sometimes very unwise on this point. What a wonder the mind of the young child is! Surely no one can have been much with little children without noticing their marvelous intuitions. You may not think, but most certainly they do think. What a mystery life is to them. Every thing is new and strange; and as they begin to reason with the first dawn of intellectual power, you must be well prepared to inform their minds, to satisfy their questioning. One thing you may be sure of: if you do not satisfy their growing desire for knowledge, some one else will do so. And will they then obtain a knowledge of good or a knowledge of evil? A child often begins to reason before he begins to speak. The process may not be comprehensible to you—certainly it is not comprehensible to the child—but it is in active existence all the same.

The great trouble, and perhaps I might say the great evil, of the present age is, people do not think. The young people of the present day are brought up, or bring themselves up, in such a different fashion from the good old home life. It seems to me sometimes, that there are no homes now. We have places to live in—we must have them; but a place to live in is not a home. I believe that much of the domestic trouble of the present day arises from or is indirectly caused by the absence of home life. A boarding house may be convenient, but it is not a home. A flat is a degree better; but a flat is not a home. Here to-day; gone to-morrow. There are no tender associations, no loving personal recollections. I do not know whether a home is most needful for the husband or for the wife. I do see, at the end of a long life of close observation of human nature, that it is needful for both, and most needful for the children.

Far better for the newly wedded to have a home, however humble, than to have a few rooms, "a place to live in," where there may be more fashionable surroundings, but where usually there will be far less mutual interest. The advantage, too, of bringing children up in a home seems almost too obvious to need comment ; yet if it were realized as it should be, there would be more home life in this country. I know the great difficulty in inducing young people to think of the future. If the present arrangement promises freedom from care, that is all they wish ; and yet how much of the future depends on the present. A love of home is almost as great a safeguard as a love of mother. The mother and the home go together as one sweetest part of early recollection. Is it not worth while to secure such memories for our dear ones?

